# Traditional Chinese medicine (*Liang-Xue-Di-Huang* Decoction) for hemorrhoid hemorrhage: Study Protocol Clinical Trial (SPIRIT Compliant)

**DOI:** 10.1097/MD.0000000000019720

**Published:** 2020-04-17

**Authors:** Shuo-Yang Shi, Qing Zhou, Zong-Qi He, Zhao-Feng Shen, Wei-Xin Zhang, Dan Zhang, Cheng-Biao Xu, Ji Geng, Ben-Sheng Wu, Yu-Gen Chen

**Affiliations:** a Nanjing University of Chinese Medicine, Changzhou Hospital of Traditional Chinese Medicine, Changzhou; b Affiliated Hospital of Nanjing University of Chinese Medicine, Nanjing; c Suzhou Hospital of Traditional Chinese Medicine, Suzhou; d Xuyi Hospital of Traditional Chinese Medicine, Huaian; e Wujin Hospital of Traditional Chinese Medicine, Changzhou, Jiangsu, China.

**Keywords:** Chinese classical prescription, hemorrhoidal disease, *Liang-Xue-Di-Huang* Decoction, randomized controlled trial

## Abstract

**Background:**

Hemorrhoidal disease (HD) is one of the commonest proctologic condition in the general population. Medical therapy for HD has not been formally confirmed due to the inconsistent of results. *Liang-Xue-Di-Huang* Decoction, a kind of ancient Chinese classical prescription, has been used to treat HD from the 19th century in China. However, clinical research of *Liang-Xue-Di-Huang* Decoction in the treatment of HD was lack. We designed this study to evaluate the efficacy and safety of *Liang-Xue-Di-Huang* Decoction in the treatment of HD.

**Methods/Design:**

A randomized, controlled, double blind, double-mimetic agent, and multicenter trial to evaluate the efficacy and safety of *Liang-Xue-Di-Huang* Decoction is proposed. HD patients (stage I, II, III) will be randomly assigned into experimental group or control group. HD patients will receive a 7-day treatments and a 7-day follow-up. The primary outcome measure is the Hemorrhoid Bleeding Score in 7 and 14 days. The Secondary outcome measures are Goligher prolapse score and quality-of-life score in 7 and 14 days.

**Discussion:**

This study will provide objective evidences to evaluate the efficacy and safety of *Liang-Xue-Di-Huang* Decoction in treatment of HD.

## 1 Introduction

Hemorrhoidal disease (HD), one of the oldest and commonest proctologic condition in the general population, is more frequent in industrialized countries.^[1]^ Chronic bleeding is the main symptom of HD.^[2]^ The true prevalence of HD in the general population is unknown and probably different from country to country.^[3]^ In the United States, HD is the third most common outpatient gastrointestinal diagnosis, affecting between 20% and 50% of the population and resulting in 4 million emergency visits annually.^[4]^

Treatments for HD include medical therapies and surgery.^[5]^ Medical therapies for HD have not been formally studied where the results have been inconsistent.^[4]^ Increased fiber and fluid intake has been shown to improve symptoms of mild-to-moderate HD bleeding.^[6]^ However, the fiber is not recommended as primary treatment to severe bleeding.^[7]^ Another common medical prescription in patients with bleeding hemorrhoids is micronized purified flavonoid fraction. These drugs include diosmin, hesperidin, and cumarin. Each of these treatments has drawbacks, such as mild gastrointestinal disturbances.^[2]^

*Liang-Xue-Di-Huang* Decoction, a kind of Chinese herbal medicine, listed in Table 1, has been used for HD from the 19th century in China with good effects and few adverse events. In 2018, with the approval of the State Administration of Traditional Chinese Medicine of the People's Republic of China, *Liang-Xue-Di-Huang* Decoction has become one of the 100 classic prescriptions that can be used directly in clinical practice.^[8]^ However, it was still necessary to prove the efficacy and safety of this ancient Chinese classical prescription.

**Table 1:**
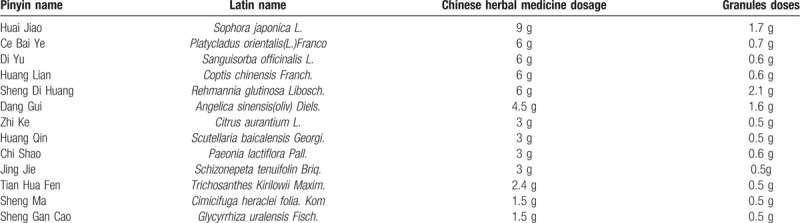
Standard formulation of *Liang-Xue-Di-Huang* Decoction.

The aim of the present study is to determine: whether *Liang-Xue-Di-Huang* Decoction is useful for relieving HD bleeding, using a randomized, controlled, blind and multicenter trial among officially registered Chinese colorectal consultants, fellows and residents in China.

## 2 Methods/design

### 2.1 Design

**Figure 1:**
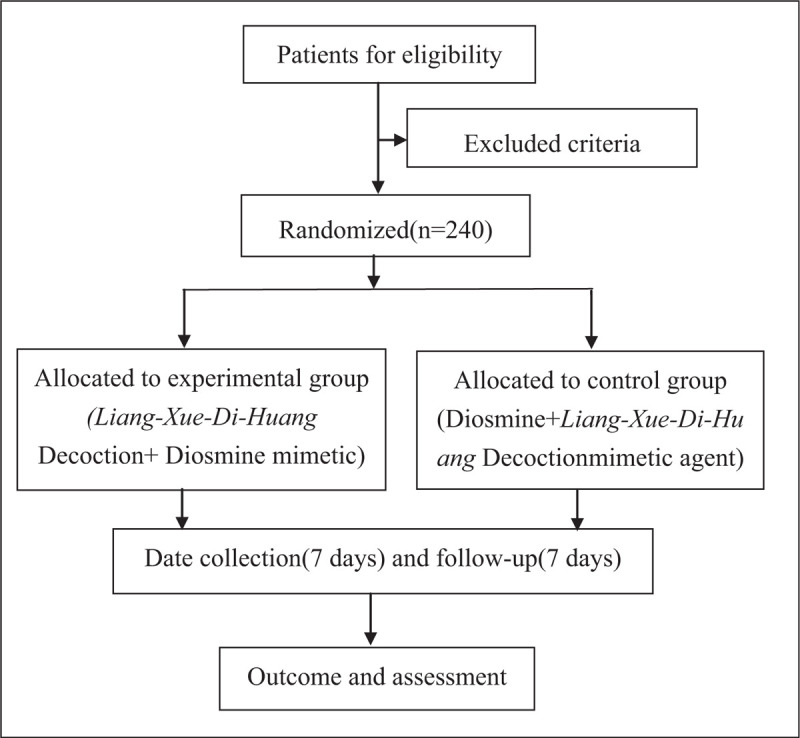
Study flow chart. The flow chart of enrolment, allocation, intervention and assessment.

This study is designed as a randomized, controlled, blind, and multicenter trial. Trained researchers introduce the trial to patients, give them information sheets and consent forms. All patients have to obtain “Ethics approval and consent to participate” section and give their written informed consents before enrolment. The study's flow chart is shown in Figure 1.

### 2.2 Ethics

The trial protocol is conducted in accordance with the Good Clinical Practice Guidelines and the Declaration of Helsinki (2008).^[9]^ Central ethical approval has been confirmed from the group leader's ethic committee of Affiliated Hospital of Nanjing University of Chinese Medicine (ref approval no. 2019NL-158-02) and 4 sub-centers ethical will comply with the group leader's ethics approval. Written informed consent will be obtained from each patient.

### 2.3 Sample size

This is a pilot study. In the relevant literature, there are no previous studies utilizing the same evaluation method as the primary outcome to compare a classic prescription.

According to the literature search results, bleeding was used as the primary outcome measure for the treatment of HD by diosmin. It was estimated that *Liang-Xue-Di-Huang* Decoction treatment is not inferior to that of diosmin treatment.

According to the non-inferiority test sample size calculation formula, for a 2-sided significance level of 0.05, the standard deviation is 0.4, (α = 0.05, β = 0.2, δ = 0.15), the non-inferiority bound is 0.15 when the degree of grasp (1−β) = 80%, the sample size is calculated using the formula:

Considering a 10% loss to follow-up, it is expected that 240 patients in over 5 centers (1:1 division into experimental group and control group) will be enrolled and followed up for at least for 7 days.

### 2.4 Recruitment

A total of 240 participants who fulfill the screening criteria will be invited to participate to this RCT at 5 hospitals in China:

1. Group leader, Affiliated Hospital of Nanjing University of Chinese Medicine, will recruit 64 participants through posters,
2. Sub-center, Changzhou Hospital of Traditional Chinese Medicine, will recruit 60 participants through posters,
3. Sub-center, Suzhou Hospital of Traditional Chinese Medicine, will recruit 60 participants through posters,
4. Sub-center, Xuyi Hospital of Traditional Chinese Medicine, will recruit 28 participants through posters,
5. Sub-center, Wujin Hospital of Traditional Chinese Medicine, will recruit 28 participants through posters.

Each-center research assistant will screen participants and obtain their written informed consent. Then, participants will be randomly allocated into either experimental group or control group. The schedule of enrolment, intervention, and assessments is detailed in Figure 1.

### 2.5 Randomization

With the help of SAS 9.4 statistical software, a random sequence will be produced by block randomization. The randomization procedure will be conducted by research assistants using an online computerized randomization system (https://sci.medroad.cn/). Before the beginning of treatment, participants were told that they have the same probability of being assigned to experimental group or control group. After the participants have completed the screening process and baseline assessment, they will be randomly assigned to experimental group or control group in a 1:1 ratio. The group numbers will be kept by an independent administrator who will not directly participate in the recruitment or follow-up of any participant.

### 2.6 Blinding

This trial is a double-blind design in which neither the patients nor the investigators (including statisticians) will be aware of their treatments during the trial period. Each hospital receives an emergency letter, which will be preserved until the end of the trial. Code-breaking should occur only in the case of serious adverse events happen, with the permission of the person in charge of the research center. A report should be submitted to the leader of the trial within 24 hours.

### 2.7 Inclusion criteria

Participants who meet all of the following criteria can be included:

1. Comply with hemorrhoids standards diagnosis (stage I, II, III)^[2]^;
  Comply with “Traditional Chinese Medicine disease and syndrome, diagnosis and curative effect standard” damp-heat syndrome: bleeding hemorrhoids, bright red blood^[10]^;
2. Participant signed the informed consent form;
3. Participant agreed to avoid other hemorrhoids medications during the trial.

### 2.8 Exclusion criteria

Participants who meet any of the following criteria will be excluded:

1. Participant had accompanied by severe liver, kidney, heart, brain, or lung dysfunction;
2. Participant had a history of inflammatory bowel disease, or a history of colorectal cancer, or had a history of any cancer;
3. Participant had a perianal abscess, anal fistula, rectal polyp, intestinal tumor, or intestinal infectious disease;
4. Participant will plan pregnancy during this study;
5. Participant is pregnant or lactating women at the time;
6. Participant was allergic to test drugs and their ingredients;
7. Participant had inability to understand the nature of the study and follow the doctor's recommendations.
8. Participant will purchase or take other hemorrhoids medications during this study period.
9. Participant had a history of bleeding disorders other than HD.

### 2.9 Test drugs

Test drugs include *Liang-Xue-Di-Huang* Decoction granules, *Liang-Xue-Di-Huang* Decoction mimetic agent granules, diosmine and diosmine mimetic agent. All the ingredients of *Liang-Xue-Di-Huang* Decoction were listed in Table 1. The total *Chinese herbal medicine dosage* in *Liang-Xue-Di-Huang* Decoction was 54.9 g per day. These herbs in *Liang-Xue-Di-Huang* Decoction were individually cooked, filtered and pressure spray dried by pharmaceutical manufacturer to form granules. Then granules were packaged in single-dose sachets. The total granules dosage of *Liang-Xue-Di-Huang* Decoction was 10.9 g per day. *Liang-Xue-Di-Huang* Decoction mimetic agent granules was consisted of 5% *Liang-Xue-Di-Huang* Decoction granules materials, as well as maltodextrin, food coloring, and bitters. *Liang-Xue-Di-Huang* Decoction granules and *Liang-Xue-Di-Huang* Decoction mimetic agent granules were provided by Tianjiang Pharmaceutical Group Co. Ltd, Wuxi, China. Diosmine mimetic agent (0.45 g, 14 tablets) was consisted of 5% diosmine materials (0.45 g, 14 tablets), as well as maltodextrin, and food coloring. Diosmine and diosmine mimetic agent were provided by Nanjing Chia Tai Tianqing Group Co. Ltd, Nanjing, China. All these mimetic agents are as close as possible in shape, size, taste, color, and package to the real drugs.

## 3 Interventions

### 3.1 Experimental group

**Figure 2:**
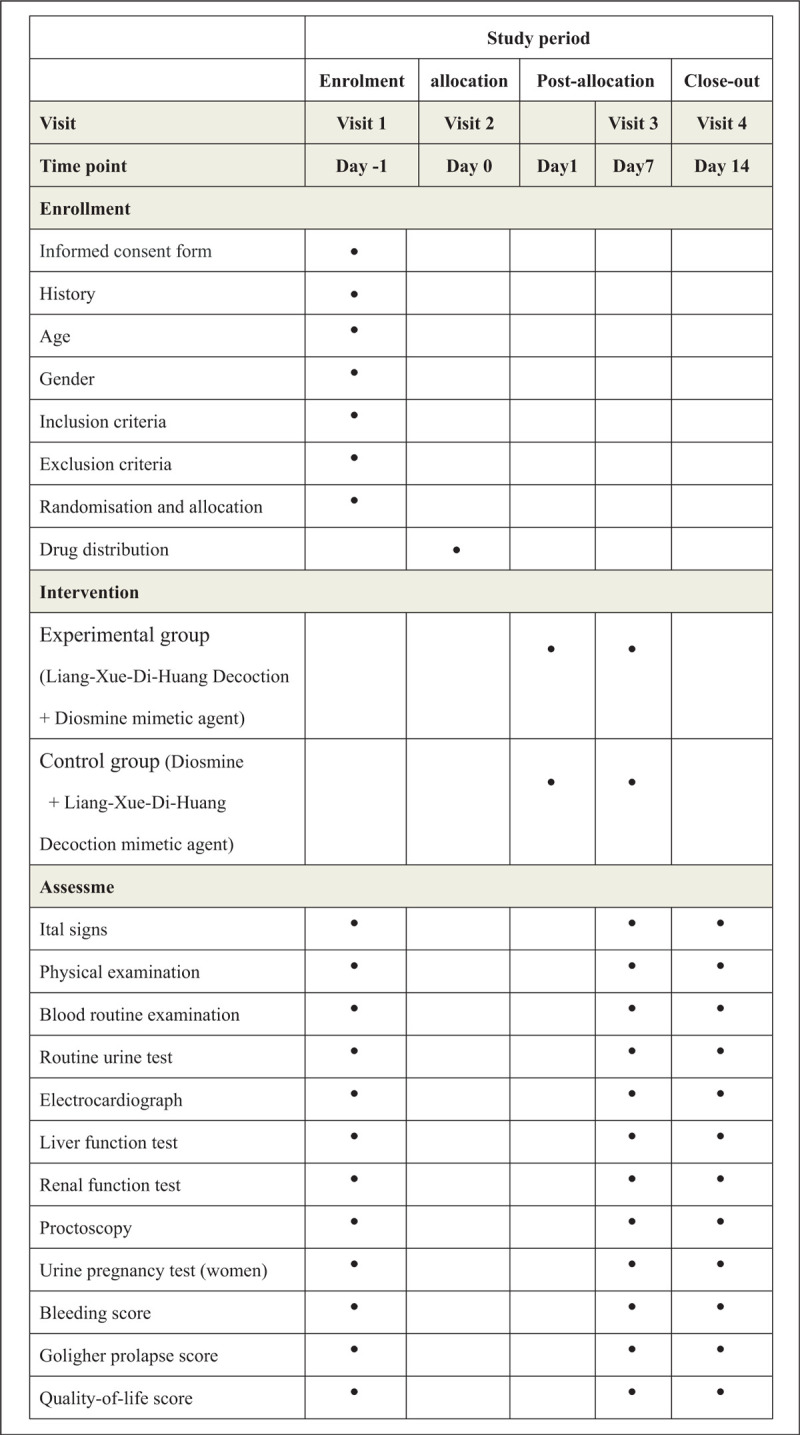
Study schedule for patients. After the enrolment and allocation, participants will receive a 7-day treatment and a 7-day follow-up. The time-points of assessment are shown in the schedule.

Participants in the experimental group will receive *Liang-Xue-Di-Huang* Decoction granules and diosmine mimetic agent. *Liang-Xue-Di-Huang* Decoction granules was 10.9 g per day, 1 hour after lunch and dinner, dissolving each dose in 100 ml warm boiled water. Diosmine mimetic agent, 0.45 g each time, 2 times a day, needs to be taken 2 hours after lunch and dinner meals. The course of treatment will last 7 days, unless there is a loss of follow-up. Patients will be followed up on the 7th and 14th days to learn about their medication and supplement use. The assessment that needs to be performed at visit is listed in Figure 2.

### 3.2 Control group

Patients in the control group will be given *Liang-Xue-Di-Huang* Decoction mimetic agent granules and diosmine. The treatments and measurements will be in accordance with experimental group.

### 3.3 Outcome measures

According to the latest treatment guidelines for HD,^[2]^ the primary outcome measure is the hemorrhoid bleeding score (Table 2). This score is a reliable and effective criterion for HD. The secondary outcome measure is Goligher prolapse score (Table 3) and quality-of-life score (Table 4).

**Table 2:**
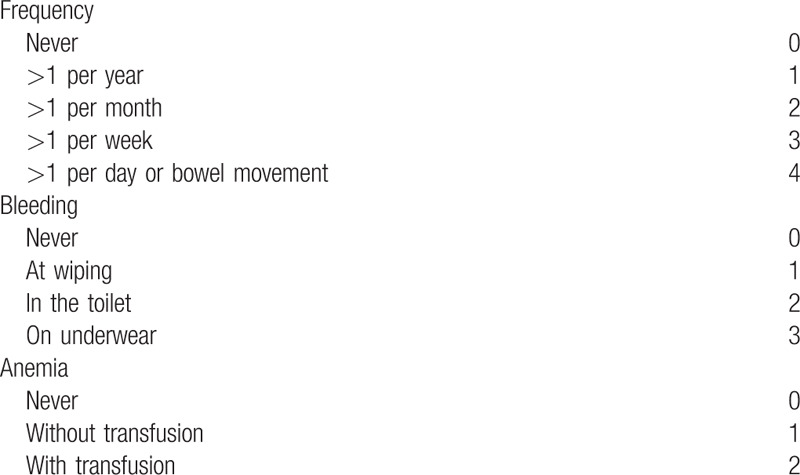
Hemorrhoid bleeding score (Possible Score = 0–9).

**Table 3:**
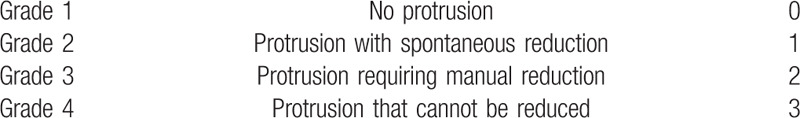
Goligher prolapse score (Possible Score = 0–3).

**Table 4:**
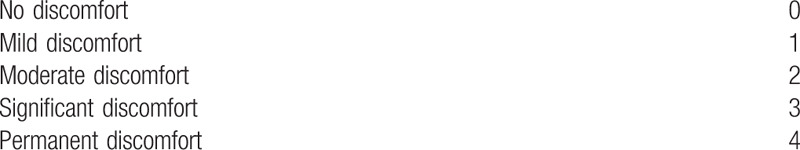
Quality-of-life score (Possible Score = 0–4).

### 3.4 Emergency symptomatic treatment (serious bleeding)

If participants have the following problems during this trial, such as a large amount of ejection bleeding accompanied by a significant decrease in hemoglobin, increased heart rate, or decreased blood pressure, participants will be stop taking these medicines and hospitalized.

### 3.5 Adverse events

Any adverse events, such as gastrointestinal reaction, liver damage, and renal failure will be recorded in each patient's case report form (CRF) irrespective of their relationship to the study intervention. When a severe adverse event occurs, this intervention will be immediately stopped and a detailed description of the time, severity, relationship with the drug will be recorded. Moreover, researchers will provide every necessary treatment, and report the adverse event to the Steering Committee and ethic committee of Affiliated Hospital of Nanjing University of Chinese Medicine within 24 hours.

### 3.6 Safety evaluation

In China, *Liang-Xue-Di-Huang* Decoction has been used from the 19th century, and the dosage used in this study is within the recommended range based on the People's Republic of China Pharmacopeia (2015 edition). Moreover, a blood routine examination, routine urine test, liver function test, renal function test, electrocardiograph, and urine pregnancy test (women only) will be administered for safety outcomes, which are monitored both before and after clinical intervention.

### 3.7 Monitoring

To ensure the quality of the trial, the monitoring data, including interim results, will be monitored by Affiliated Hospital of Nanjing University of Chinese Medicine and Jiangsu Famous Medical Technology Co., Ltd, Nanjing, China. If any issues are found, the group leader can decide to change this agreement. In addition, Jiangsu Famous Medical Technology's clinical trial expert will supervise this clinical study. The lead researcher will be solely responsible for conducting the trial and make the final decision on any changes required.

### 3.8 Data management

Information from the clinical examination, as well as evaluation of treatment efficacy, will be recorded in each patient's CRF. The study record is the source document of clinical study subject and should be kept in group leader's hospital. Each center will design designated personnel to be the electronic CRF input staff. Upon completion of each subject observation, the investigator will promptly submit the study record to the CRF inputter within 1 week. The electronic CRF encoder must review whether the project record of the study notes is complete and report on time. The clinical supervisor will monitor the work of the clinical trial center at least once a month.

### 3.9 Patient's privacy protection

Only researchers and arbitrator who will sign the confidentiality commitment in this clinical trial may come into contact with the participants’ personal health records. Drug regulatory departments have the right to inspect the records of clinical trials. Data will be processed anonymously, and information identifying individual subjects will be omitted. Patient's medical records will be stored in group leader's data archives.

### 3.10 Statistical analysis

Frequency, median, and mean ± standard deviation of the bleeding score, Goligher prolapse score and quality-of-life score will be used for descriptive statistics. The statistical analysis will be performed using SAS 9.4. *P* < .05 is considered statistically significant.

## 4 Discussion

HD is a well-defined clinical and pathophysiological placement between benign conditions, but with high impact on quality of life.^[11]^ Bleeding is the main symptom of HD.^[4]^ It is estimated that 50% of people over 50 years of age have experienced symptoms of HD at least once in their life, and one-third of patients affected by HD seek medical attention.^[12,13]^ Excessive bleeding can lead to an emergency situation. Hemorrhoidectomy and stapled hemorrhoidopexy are validated and effective surgical techniques, but are associated with long, painful postoperative courses.^[14]^ Contrary to common belief, a nonsurgical treatment is quite effective to manage HD,^[7]^ which can be offered with expectations of minimal harm.^[6]^ Therefore, medical therapy should be the first-line therapy for this disease.^[5]^

*Liang-Xue-Di-Huang* Decoction has been used to treat HD from the 19th century in China. Rich experience has been accumulated. In 2018, National Administration of Traditional Chinese Medicine of People's Republic of China published 100 classic prescriptions of ancient Chinese medicine, which can be used directly in clinical practice in China. *Liang-Xue-Di-Huang* Decoction was one of 100 classic prescriptions of ancient Chinese medicine. In this classic *Liang-Xue-Di-Huang* Decoction, *Sophora japonica L.* (Huai Jiao) and *Platycladus orientalis(L.)Franco* (Ce Bai Ye) were the Jun (emperor) components. *Sophora japonica L.* (Huai Jiao), which was first recorded in Shen Nong's herbal classic, was commonly applied in clinical practice for the treatment of hematochezia from the 1th century in China. It has the effect of cooling blood, stopping bleeding, clearing heat in bowels and eliminating swell and easing pain.^[15]^ Modern pharmacological studies have demonstrated its efficacy for stopping bleeding and anti-inflammation.^[16]^*Platycladus orientalis(L.)Franco* (Ce Bai Ye) was categorized as a blood cooling and hematostatic herb, which was usually prescribed with heat-clearing herbs to reinforce the efficacy of hemostasis. *Sanguisorba officinalis L.* (Di Yu), *Coptis chinensis Franch*. (Huang Lian), *Rehmannia glutinosa Libosch.* (Sheng Di Huang) were the Chen (minister) components, synergize with Jun to strengthen its therapeutic effects. In traditional Chinese medicine, *Sanguisorba officinalis L.* (Di Yu) was often mixed with other herbs for the treatment of bleeding hemorrhoids. *Coptis chinensis Franch*. (Huang Lian) had the effect of detumescence, clinically used for the treatment of hemorrhoid. *Rehmannia glutinosa Libosch.* (Sheng Di Huang) has been traditionally used as a blood cooling hemostatic. The Zuo (assistant) components, *Angelica sinensis(oliv) Diels.* (Dang Gui), *Citrus aurantium L.* (Zhi Ke), *Scutellaria baicalensis Georgi.* (Huang Qin), *Paeonia lactiflora Pall.* (Chi Shao), *Schizonepeta tenuifolin Briq.* (Jing Jie), and *Trichosanthes Kirilowii Maxim* (Tian Hua Fen), activated blood circulation to remove stasis, eliminated possible adverse effects of the Jun and/or Chen components. The Shi (courier) components, *Cimicifuga heraclei folia.Kom* (Sheng Ma), and *Glycyrrhiza uralensis Fisch.* (Sheng Gan Cao) facilitated the overall action of the other components. Theoretically, *Liang-Xue-Di-Huang* Decoction worked through the traditional Chinese medicine therapeutic principle“Jun-Chen-Zuo-Shi”, to relieve bleeding hemorrhoids and diminish hemorrhoids prolapsed.

Although traditional Chinese medicine has been clinically practiced for thousands of years, most Chinese herbal medicine products do not possess up-to-date regarding about their safety and modern scientific evidences for their claimed clinical uses. To the best of our knowledge, this is the first clinical study to investigate the efficacy and safety of *Liang-Xue-Di-Huang* Decoction in the treatment of HD. If this study confirms that *Liang-Xue-Di-Huang* Decoction is effective and safe, *Liang-Xue-Di-Huang* Decoction could be implemented as a new medical therapy to relieve hemorrhoid bleeding in China and Western countries.

## Author contributions

Shuo-Yang Shi and Qing Zhou contributed to the design of the study protocol. Zhao-feng Shen participated in the statistical design and helped in the design of the study. Wei-Xin Zhang, Dan Zhang, Ben-Sheng Wu, Cheng-Biao Xu, Ji Geng, and Zong-Qi He helped to draft the manuscript and participated in the project development. Yu-gen Chen was the project leader for this research and participated in the critical revision of the manuscript. All authors have read and approved the final manuscript.
